# The N13 somatosensory evoked potential in complex regional pain syndrome – A potential marker for central sensitization?

**DOI:** 10.1016/j.cnp.2026.06.013

**Published:** 2026-07-01

**Authors:** Florin Allmendinger, Martin Schubert, Florian Brunner, Caterina Leone, Michèle Hubli

**Affiliations:** a Spinal Cord Injury Center, Balgrist University Hospital, University of Zurich, Zurich, Switzerland; b Department of Physical Medicine and Rheumatology, Balgrist University Hospital, University of Zurich, Zurich, Switzerland; c Department of Human Neuroscience, Sapienza University of Rome, Rome, Italy; d Neuroscience Center Zurich (ZNZ), University of Zurich and ETH, Zurich, Zurich, Switzerland

**Keywords:** Complex regional pain syndrome, Central sensitization, Hyperalgesia, Somatosensory evoked potentials, Spinal hyperexcitability

## Abstract

**Objective:**

The cervical N13 component of upper-limb somatosensory evoked potentials (SEP) is thought to arise from segmental, post-synaptic dorsal horn neurons. Recent studies have shown that N13 SEPs were increased after experimentally-induced central sensitization in healthy individuals, suggesting its potential as a readout of spinal hyperexcitability. This study aimed to assess the clinical utility of N13 SEPs in individuals with complex regional pain syndrome (CRPS), showing pronounced signs of central sensitization.

**Methods:**

Ten individuals with CRPS and 10 healthy controls (HC) were enrolled in this pilot study. Sensory function was well characterized and multilevel SEPs (i.e., N9, N13 and N20/P25) were recorded bilaterally after electrical stimulation of the median nerve.

**Results:**

Although the CRPS cohort showed signs of central sensitization (i.e., mechanical hyperalgesia and increased temporal pain summation) there was no difference in N13 amplitudes between the two cohorts (*p* = 0.53).

**Conclusion:**

Our findings challenge the use of N13 SEPs as a reliable marker of increased dorsal horn excitability in CRPS. Additional influences, including peripheral sensitization and medication effects may mask potential differences and need to be considered in future applications.

**Significance:**

This study highlights the obstacles of translating neurophysiological markers of central sensitization from experimental models into clinical pain cohorts.

## Highlights

• Evaluation of N13 somatosensory evoked potentials (SEP) as marker for sensitization.
• No increase in N13 SEP amplitudes in complex regional pain syndrome (CRPS).
• N13 SEP amplitudes do not reflect the magnitude of hyperalgesia in CRPS.

## 1 Introduction

The cervical N13 component of upper-limb somatosensory evoked potentials (SEP) is mediated by non-nociceptive Aβ-fibers and reflects postsynaptic activity within the cervical spinal cord (Stöhr, 2005). Electrophysiological studies suggest that the generator of the posterior cervical N13 component is located within deeper laminae of the cervical dorsal horn (Desmedt and Nguyen, 1984; Jeanmonod et al., 1991), consistent with experimental findings obtained from intraspinal recordings in animal models (Beall et al., 1977; Coombs et al., 1956). Recent studies have demonstrated that spinally-generated N13 amplitudes increase after experimentally-induced central sensitization (i.e., induced with topical capsaicin or low frequency stimulation (LFS)) in healthy participants (Di Lionardo et al., 2021; Leone et al., 2023) and therefore might serve as a marker for dorsal horn excitability, reflecting the activity of wide dynamic range neurons (Leone et al., 2025). Two other observations support this notion. First, the facilitation of N13 SEPs after sensitization with capsaicin could be prevented with the administration of pregabalin (Di Lionardo et al., 2021) – a drug acting on spinal dorsal horn neurons (Tuchman et al., 2010). Second, N13 SEPs have been shown to be modulated by a noxious conditioning stimulus, i.e., cold water (Di Pietro et al., 2021), also acting on dorsal horn transmission (Crawford et al., 2022; Leone and Truini, 2019).

Central sensitization refers to increased responsiveness of nociceptive neurons in the central nervous system to normal or subthreshold input (*IASP Terminology*, n.d.) and is a key mechanism underlying the development of chronic pain (Arendt-Nielsen et al., 2018). Sensitization of the central nervous system is also thought to play an essential role in the pathophysiology of complex regional pain syndrome (CRPS) (Eldufani et al., 2020; Ferraro et al., 2024; Marinus et al., 2011), leading to hypersensitivities within the affected limb (De Schoenmacker et al., 2023; Drummond et al., 2018; Reimer et al., 2016) and even initially unaffected areas (Maleki et al., 2000; Van Rijn et al., 2011). With the pronounced mechanical hyperalgesia and allodynia, CRPS poses an excellent clinical model for central sensitization. To what extent these hypersensitivities are driven by spinal hyperexcitability is, however, challenging to investigate in humans since spinal neurons cannot be assessed by direct electrophysiological recordings, but non-invasive neurophysiological approaches are needed to assess these central nervous system changes (Arendt-Nielsen et al., 2018).

Hence, this is the first study exploring the clinical application of N13 SEPs in the context of central sensitization in chronic pain patients. We investigated whether the cervical N13 SEP component can serve as a potential marker of spinal hyperexcitability in individuals with CRPS with pronounced signs of central sensitization, i.e., allodynia and hyperalgesia. Multilevel SEPs (i.e., peripheral N9, spinal N13, cortical N20/P25) were compared between individuals with CRPS and healthy controls (HC), as well as between the affected and non-affected limbs within the cohort of CRPS. In addition, we investigated the association of psychophysical measures of hypersensitivity with N13 SEPs in the cohort of CRPS. We hypothesized that N13 SEP amplitudes are increased after stimulation of the painful hand of CRPS compared to HC and compared to their contralateral hand.

## 2 Methods

### 2.1 Participants

Individuals with hand CRPS type I were recruited by an experienced rheumatologist (F.B.) at the Department of Physical Medicine and Rheumatology of the Balgrist University Hospital in Zurich, Switzerland, or from the patient organization CRPS Schweiz. Individuals included in the study had to be diagnosed with CRPS type I, were between 18 and 80 years old, were affected at only one upper extremity, suffered from ongoing pain and showed clear signs of central sensitization, i.e., mechanical hyperalgesia and/or allodynia. Exclusion criteria were pregnancy and any neurological disorder or history of chronic pain other than CRPS-related pain.

In addition, age- and sex-matched HC were recruited. Exclusion criteria for HC were the same as for the individuals with CRPS with the addition of no acute pain or the intake of pain medication.

Written informed consent was obtained from all individuals prior to the study. The study was approved by the local ethics committee “Kantonale Ethikkommission Zürich” (2024–00134, clinicaltrials.gov number: NCT06443281) and conducted in accordance with the Declaration of Helsinki.

### 2.2 Study design

The study consisted of a single two-hour visit. The visit comprised the evaluation of pain characteristics, the assessment of the CRPS severity score (CSS) (Harden et al., 2010), nerve conduction study (NCS), quantitative sensory testing (QST), and multilevel SEPs. Pain characteristics and CSS were only assessed in individuals with CRPS. NCS, QST and SEPs were always performed on the painful and the contralateral, pain-free hand. In HC, both arms were examined and were either designated as “painful arm” or “contralateral arm” based on matching laterality (left/right) to that of the corresponding individual with CRPS.

### 2.3 Pain characteristics

Pain characteristics included the assessment of pain intensity on a numeric rating scale (NRS) on the day of the study, the average pain over the last seven days and the maximum pain intensity reached within these seven days, as well as the intake of regular pain medication. Further, to determine the spatial pain extent, individuals with CRPS filled out a pain drawing where they marked their painful areas on standardized body charts (including dorsal and frontal view). These pain drawings were scanned, digitalized and further analyzed with a custom-made algorithm to calculate the percentage of marked pixels to the total amount of pixels of the full body (Rosner et al., 2021).

### 2.4 Nerve conduction study

Orthodromic sensory nerve conduction of the median nerve was measured to ensure peripheral nerve integrity. Measurements were performed using routine electrodiagnostic equipment (Keypoint G4 system, Natus Medical Incorporated, Pleasanton, CA, USA) and were assessed according to clinical standard (Stöhr, 2014). A block electrode was used to stimulate over the second digit (stimulation duration: 0.2 ms; stimulation frequency: 2.1 Hz; high-pass filter: 50 Hz; low-pass filter: 1000 Hz). Supramaximal stimulation intensity (i.e., higher than the intensity required for the maximal sensory action potential) was used. All subjects were examined in an upright seated position with the forearm resting on their lap. Recording electrodes (BlueSensors, Ambu, Denmark) were placed over the median nerve at the ventral part of the wrist, approximately 2 cm from the wrist crease. A minimum of 15 recorded stimuli were averaged for the analysis. Minimum and maximum of the amplitude were determined directly on the recording device. Amplitude and nerve conduction velocity (NCV) were calculated for both arms.

NCS were performed bilaterally for all but three participants (2 CRPS, 1 HC). In these first three participants, only one side was assessed (painful side in CRPS; matched side in HC).

### 2.5 Quantitative sensory testing and temporal summation of pain

A subset of the QST protocol was performed according to the definition of the German Research Network on Neuropathic Pain (DFNS) (Rolke et al., 2006) to detect potential mechanical and thermal hypersensitivities. This subset comprised the evaluation of heat pain thresholds (HPT), mechanical pain thresholds (MPT), stimulus-response function (SR-function, including mechanical pain sensitivity (MPS) and dynamic mechanical allodynia (DMA)), and pressure pain thresholds (PPT). All participants were familiarized with each measurement in a pain-free area, i.e., the contralateral forearm. The recorded QST data were normalized to reference values using the eQuiSTA software (version 1.3.7) provided by the DFNS and are presented as z-scores, where positive values indicate a sensory gain of function and negative values a sensory loss of function relative to reference data.

Additionally, temporal summation of pain (TSP) was assessed in response to twelve consecutive pinprick stimuli (MRC Systems, Heidelberg, Germany) applied to the painful and the contralateral hand area at a frequency of 0.33 Hz. The intensity of the pinprick stimuli was matched to an NRS 4 out of 10, with an upper limit of stimulation intensity at 512 mN. During the TSP protocol, participants rated each stimulation on the NRS. The mean pain rating of the first three and the last three pinprick stimuli was calculated after each assessment. Further, the increase in pain ratings from the first three to the last three stimuli was calculated and used for further analysis.

### 2.6 Somatosensory evoked potentials

Multilevel, i.e., peripheral, spinal, and cortical, SEPs were recorded with the Keypoint G4 system after electrical stimulation of the median nerve (block electrode; stimulation duration: 0.2 ms; stimulation frequency: 3.1 Hz; high-pass filter: 2 Hz; low-pass filter: 2000 Hz) at the wrist. The stimulation intensity was set to 3 mA above the motor threshold (i.e., the lowest intensity inducing a visible thumb twitch) for each individual and arm. Participants lay in a supine position with their eyes closed. Two runs of 500 trials each were collected and averaged.

The placement of the recording electrodes followed the International Federation of Clinical Neurophysiology guidelines (Cruccu et al., 2008). The peripheral N9 component was recorded with disposable surface electrodes (BlueSensors, Ambu, Denmark) over the Erb point bilaterally, 2–3 cm above the clavicle, within the angle formed by the posterior border of the clavicular head of the sternocleidomastoid muscle and the clavicle. The electrode ipsilateral to the stimulation side was hereby the active recording electrode, while the contralateral electrode functioned as reference electrode. The spinal N13 component was recorded with disposable surface electrodes (BlueSensors, Ambu, Denmark) placed over the sixth cervical spinous process (active) and the anterior neck at the level of the glottis. The cortical N20 and P25 components were recorded using disposable needle electrodes (Neurodart, Spes Medica, Italy). The active electrode was placed contralateral to the side of stimulation at positions C3’/C4’ with a reference electrode placed over Fz, according to the international 10–20 electroencephalogram recording system. Impedance of all electrodes was kept below 5000 Ω.

The individual multilevel SEPs were extracted and analyzed offline. A customized algorithm in RStudio (R version 4.2.3 for Windows) was used for automated peak detection of the different SEP components. Detected peaks underwent visual inspection to ensure correct detection. N9 amplitude was measured between the N9 and the preceding P8 peak. N13 amplitude was measured between the N13 and the preceding P9 peak. N20/P25 amplitude was measured between the N20 and the following P25 peak.

### 2.7 Statistical analyses

Statistical analyses were performed in R statistical software (R version 4.2.3 for Windows).

Differences in age, height and weight between individuals with CRPS and HC were compared using unpaired *t*-tests or Wilcoxon rank-sum tests, depending on normality of the data distribution (Shapiro-Wilk tests).

Separate linear mixed models (‘lmer’ function of the R package ‘lme4’) were used to assess the main effect of ‘cohort’ (CRPS and HC) and ‘area’ (painful and contralateral) on outcomes of the NCS (amplitude and NCV), QST z-scores (HPT, MPT, MPS and PPT), the stimulation intensity used during the assessment of SEPs, as well as the amplitudes of the different SEP components (N9, N13 and N20/P25). The interaction term ‘cohort x area’ and the random effect ‘Participant-ID’ was included in all models.

Two additional linear mixed models were used to assess the main effect ‘cohort’ and ‘stimulation’ (stimulation 1–3 and stimulation 10–12) on the pain ratings of the TSP on the painful and the contralateral hand. The interaction term ‘cohort x stimulation’ and the random effect ‘Participant-ID’ was included in all models.

Post-hoc multiple comparisons (R package ‘emmeans’) were performed on significant interaction terms or, in absence of significant interaction, on significant main effects. In line with our hypotheses, post-hoc contrasts on ‘cohort x area’ were limited to between-cohort comparisons within each area and to area differences within the CRPS cohort. No area contrasts were performed in HC, as no differences were anticipated. Effect sizes were calculated as Hedges' g (R package ‘effsize’) for all post-hoc comparisons. Full model outputs and detailed post-hoc comparisons are provided in supplementary Tables S1–S3.

The presence of DMA was compared between cohorts (painful limb) using Fisher's exact test, and within the CRPS cohort (painful vs contralateral limb) using McNemar's test.

Lastly, Spearman correlations were used to test the association of the N13 amplitude after stimulation of the painful arm of individuals with CRPS with QST parameters (HPT, MPT, MPS, PPT, DMA, and TSP).

Fulfillment of model criteria were checked with diagnostic plots (i.e., quantile-quantile plots and histograms). Statistical tests were performed at an α level of 0.05. To control for multiple testing, *p*-values were adjusted for all comparisons using the Benjamini–Hochberg procedure.

## 3 Results

### 3.1 Demographics and CRPS characteristics

**Fig. 1:**
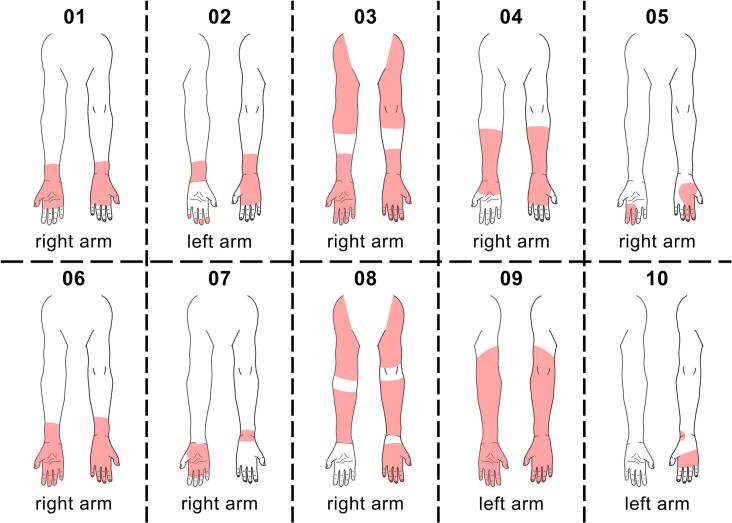
Dorsal (right) and frontal (left) CRPS-specific pain extent (red area) within the painful limb of individuals with CRPS. (For interpretation of the references to colour in this figure legend, the reader is referred to the web version of this article.)

Ten female individuals with CPRS and ten female HC were recruited. There were no significant differences in age (CRPS: 37.6 ± 12.9 years, HC: 37.9 ± 13.1 years, W = 49.5, *p* = 1.0) and height (CRPS: 171 ± 5 cm, HC: 167 ± 7 cm, *t* = 1.25; *p* = 0.38) between the two cohorts. However, individuals with CRPS (77.7 ± 12.5 kg) were significantly heavier than HC (60.0 ± 7.3 kg, *t* = 3.87, *p* = 0.004). CSS, CRPS-specific pain characteristics, and medication intake are presented in Table 1. In total, nine individuals with CRPS (90%) reported using at least one pain medication. The CRPS-specific pain extent is shown in Fig. 1.

**Table 1:** CRPS characteristics and medication intake.

Pain characteristics	Mean ± SD (range)
Pain current [NRS]	4.4 ± 1.9 (2–8.5)
Average pain last 7 days [NRS]	4.9 ± 2.0 (2–8.5)
Maximum pain last 7 days [NRS]	8.2 ± 2.3 (2.5–10)
Pain duration [months]	47 ± 38 (6–100)
CRPS severity score [0–17]	13.1 ± 2.4 (9–16)
Spatial pain extent [% body surface]	5.1 ± 5.2 (0.7–15.4)

Medication	N (%)
NSAID	4 (40)
Cannabinoids	1 (10)
Opioids	2 (20)
Antidepressants	4 (40)
Antiepileptics	4 (40)

*Abbreviations: CRPS: complex regional pain syndrome; NRS: numeric rating scale; NSAID: non-steroidal anti-inflammatory drugs*.

### 3.2 Nerve conduction study

Mean ± SD of the amplitudes and NCV of the NCS assessed in the painful and contralateral limb of CRPS and HC can be found in the supplementary Table S4.

Amplitudes and NCV of all recorded median neurographies were within the range of published reference values of healthy individuals (Chouhan et al., 2021) and above clinical cut-off values for amplitudes (<6.9 uV) and NCV (<46.9 m/s) of sensory median neurographies (Stöhr, 2014). There was no significant interaction ‘cohort x area’ (F = 0.06, *p* = 0.82), nor a significant main effect of ‘cohort’ (F = 2.02, *p* = 0.17) or ‘area’ (F = 4.42, *p* = 0.052) on amplitudes. In contrast, there was a significant interaction ‘cohort x area’ (F = 8.30, *p* = 0.01) on NCV. However, post-hoc comparisons revealed that NCV did not significantly differ between CRPS and HC, neither assessed in the painful limb (CRPS: 57.2 ± 3.4 m/s, HC: 57.2 ± 3.4 m/s, *t* = −0.03, *p* = 1.0 Hedges' g = −0.01) nor in the contralateral limb (CRPS: 56.7 ± 3.7 m/s, HC: 59.0 ± 4.0 m/s, *t* = −1.57, *p* = 0.24, Hedges' g = −0.55). Further, NCV did not significantly differ between the painful and the contralateral limb in CRPS (*t* = 1.12, *p* = 0.41, Hedges' g = 0.19).

### 3.3 Quantitative sensory testing

**Fig. 2:**
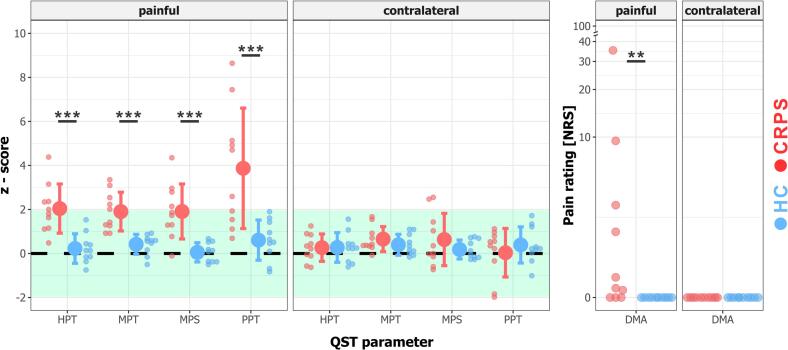
QST z-scores (mean ± SD) of the heat pain threshold (HPT), mechanical pain threshold (MPT), mechanical pain sensitivity (MPS) and pressure pain threshold (PPT), as well as pain ratings during assessment of dynamic mechanical allodynia (DMA), of the painful and the control area in individuals with CRPS and HC. **p < 0.01; ***p < 0.001.

Mean ± SD of the different QST parameters measured in the painful and contralateral limb of CRPS and HC respectively are illustrated in Fig. 2 and can be found in the supplementary Table S5.

HPT, MPT, MPS and PPT were different between the two cohorts and measured areas (‘cohort x area’; HPT: F = 16.36, *p* < 0.001; MPT: F = 13.74, *p* = 0.002; MPS: F = 16.34, p < 0.001; PPT: F = 13.41, p = 0.002). Post-hoc comparisons showed that, in the painful limb, the cohort of CRPS showed pronounced thermal and mechanical hyperalgesia compared to HC as well as compared to their contralateral limb (all p < 0.001; supplementary Table S3). Further, DMA was present in the painful limb of seven individuals with CRPS (70%) but in none of the HC (Fisher's exact test, *p* = 0.003). None of the QST parameters significantly differed between the contralateral limb of CRPS and HC (all *p* > 0.41).

**Fig. 3:**
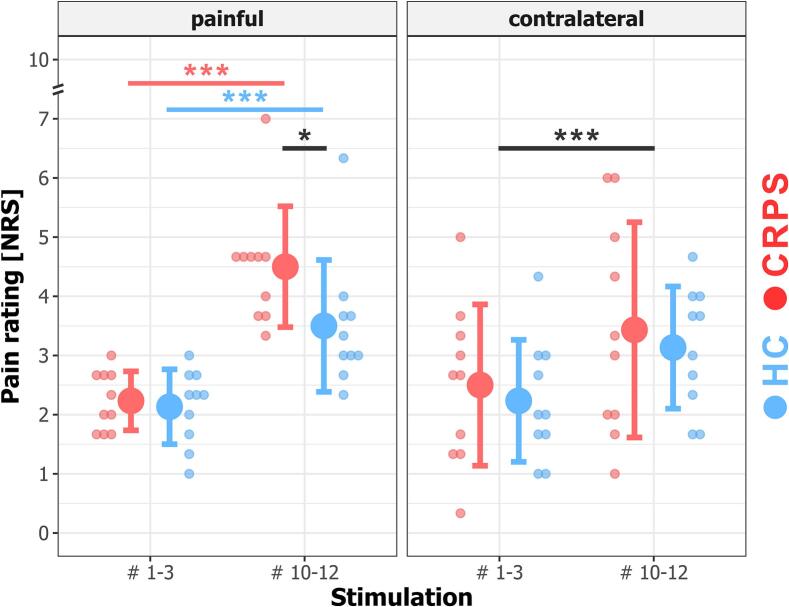
Temporal summation of pain in the painful and the contralateral area in individuals with CRPS and HC depicted as mean ± SD of the pain ratings of the first three (#1–3) and last three (#10–12) stimuli. *p < 0.05; ***p < 0.001.

A significant interaction effect was found for the TSP assessed in the painful limb (‘cohort x stimulation’, F = 4.67; *p* = 0.04). Both, individuals with CRPS (stim. 1–3: 2.2 ± 0.5, stim. 10–12: 4.5 ± 1.0, *t* = 7.70, p < 0.001, Hedges' g = 2.34) and HC (stim. 1–3: 2.1 ± 0.6, stim. 10–12: 3.5 ± 1.1, *t* = 4.64, p < 0.001, Hedges' g = 1.29) showed a pronounced TSP (Fig. 3). However, individuals with CRPS presented with higher TSP than HC (*t* = 2.61, *p* = 0.03, Hedges' g = 0.90).

In the contralateral limb, only a significant effect of ‘stimulation’ (F = 18.43, p < 0.001) was found, showing that, independent of cohort, there was a pronounced TSP observed (*t* = 8.72, p < 0.001, Hedges' g = 0.64) (Fig. 3).

### 3.4 Multilevel somatosensory evoked potentials

There was no significant interaction between cohort and area for used stimulation intensities (‘cohort x area’: F = 0.02, *p* = 0.90), nor a significant main effect of ‘cohort’ (F = 0.02, p = 0.90) or ‘area’ (F = 2.66, *p* = 0.12).

**Fig. 4:**
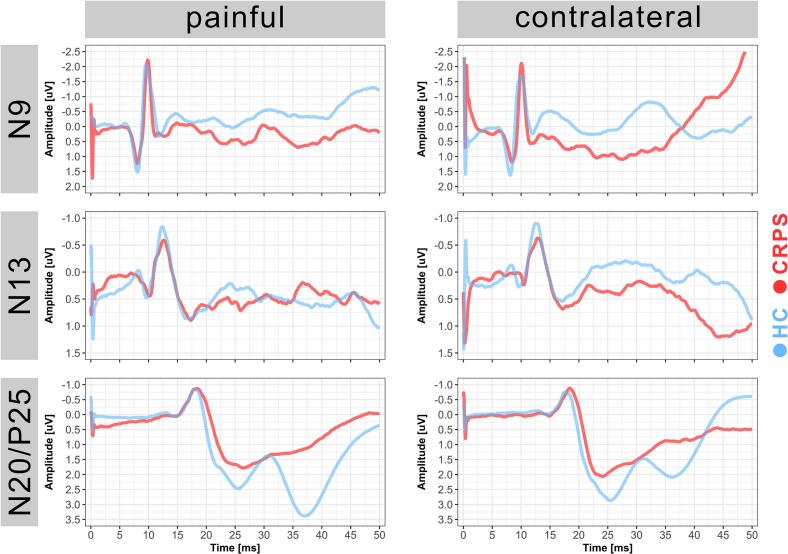
Grand average of multilevel SEPs (N9, N13, N20/P25) of both cohorts (CRPS, HC) after stimulation of the painful and the contralateral arm.

**Fig. 5:**
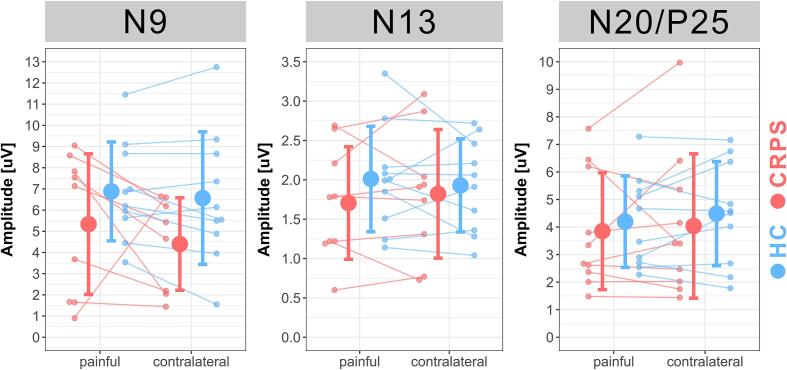
Mean ± SD of amplitudes of the multilevel SEPs (N9, N13, N20/P25) of both cohorts (CRPS, HC) after stimulation of both the painful and the contralateral arm.

Due to a high number of artefacts, three N9 components (1 CRPS, both sides; 1 CRPS, contralateral side) and one N13 component (1 CRPS, contralateral side) could not be analyzed. Further, one individual with CRPS did not present a N13 component after stimulation of the painful arm. Fig. 4 shows the grand averages of the different SEP components of both cohorts and assessed arms. Mean ± SD of SEP latencies and amplitudes, as well as the used stimulation intensity can be found in Table 2, and the SEP amplitudes are additionally illustrated in Fig. 5. The latencies of all recorded SEPs were within normal range (N9, <12.4 ms; N13, <15.8 ms; N20, <22.3 ms) (Stöhr et al., 2005).

**Table 2:** Mean ± SD of latencies and amplitudes of the N9, N13 and N20/P25 SEP components.

area	cohort	SEP component		Latency [ms]	Amplitude [uV]
painful	CRPS	N9	*N* = 9	9.87 ± 0.63	5.34 ± 3.31
		N13	N = 9	12.88 ± 0.79	1.71 ± 0.71
		N20/P25	*N* = 10	18.80 ± 1.08	3.85 ± 2.12
	HC	N9	N = 10	9.73 ± 0.72	6.88 ± 2.33
		N13	N = 10	12.55 ± 0.63	2.01 ± 0.67
		N20/P25	N = 10	18.34 ± 0.95	4.19 ± 1.66

contralateral	CRPS	N9	*N* = 8	10.03 ± 0.62	4.40 ± 2.18
		N13	N = 9	12.97 ± 0.69	1.82 ± 0.82
		N20/P25	N = 10	18.78 ± 18.78	4.04 ± 2.62
	HC	N9	N = 10	9.72 ± 0.76	6.57 ± 3.13
		N13	N = 10	12.75 ± 0.61	1.93 ± 0.59
		N20/P25	N = 10	18.23 ± 0.95	4.49 ± 1.89

*Abbreviations: CRPS: complex regional pain syndrome; HC: healthy controls; SD: standard deviation; SEP: somatosensory evoked potential*.

The analyses did not reveal a significant interaction between cohorts and area for N13 amplitudes (‘cohort x area’: F = 0.42, *p* = 0.53), nor for the other SEP amplitudes (N9: F = 0.72, *p* = 0.41; N20/P25: F = 0.03, *p* = 0.86). No significant main effects of ‘cohort’ or ‘area’ were found for any of the three components (all *p* > 0.10).

### 3.5 Association of N13 amplitude and signs of hypersensitivity

The N13 amplitude recorded after stimulation of the painful arm of individuals with CRPS was not significantly correlated with any of the QST parameters (HPT: rho = −0.56, *p* = 0.23; MPT: rho = −0.03, *p* = 1.0; MPS: rho = −0.25, *p* = 0.66; PPT: rho = −0.34, *p* = 0.49; DMA: rho = 0.49, *p* = 0.31) measured in the painful area. Further, the N13 amplitude also did not correlate with TSP (rho = 0.06, p = 1.0), a common psychophysical measure of spinal excitability (Chen et al., 2025).

## 4 Discussion

The aim of this study was to investigate the use of the N13 SEP component as a potential marker of central sensitization in a cohort of individuals with CRPS type I. Despite the successful inclusion of individuals with CRPS and pronounced signs of central sensitization, i.e., mechanical hyperalgesia, the N13 amplitudes neither differed from those of HC, nor between the painful and contralateral side within the cohort of CRPS. In the following paragraphs, we will discuss potential reasons why no facilitation of N13 amplitudes was observed in CRPS and its implications for the interpretation of the N13 SEP as a potential clinical marker of central sensitization.

### 4.1 Peripheral and central changes in CRPS

As the pathophysiology of CRPS involves alterations in both the peripheral and the central nervous system (Eldufani et al., 2020; Ferraro et al., 2024; Marinus et al., 2011), one possible explanation for the absence of increased N13 amplitudes in CRPS could be that the pain observed in our CRPS cohort may not be predominantly driven by central sensitization. In line with previous studies (Allmendinger et al., 2024; Drummond et al., 2018; Reimer et al., 2016), our CRPS cohort was characterized by pronounced mechanical and thermal hyperalgesia, as well as allodynia within the painful limb. While mechanical hyperalgesia and allodynia are clear signs of central sensitization, thermal and pressure hyperalgesia can be linked to peripheral sensitization (Jensen and Finnerup, 2014; Treede et al., 2022; Vollert et al., 2018). Therefore, the individuals included in this study manifested signs of ongoing peripheral sensitization. However, in addition to mechanical hyperalgesia and the presence of allodynia, our results also showed increased TSP in the affected limb of CRPS, compared to HC, which replicates previous findings of ours (De Schoenmacker et al., 2023). The phenomenon of TSP is considered to represent the excitability of second-order neurons in the spinal dorsal horn, and is therefore regarded as a purely central process (Mendell and Wall, 1965; Price and Dubner, 1977). Hence, alongside the observed peripheral sensitization, we would argue that central sensitization, specifically hyperexcitability of second-order neurons, such as wide dynamic range neurons, contributes to the pain phenotype observed in our cohort. Because a non-cephalic reference during N13 SEP assessment isolates purely spinal processes (Desmedt and Nguyen, 1984), the measure specifically reflects spinal activity. Despite this spinal hyperexcitability being psychophysically evident in enhanced TSP, we did not observe corresponding neurophysiological increases in N13 amplitudes, creating a discrepancy that requires further explanation.

Currently, evidence that N13 SEPs capture the increase in spinal excitability stems solely from experimental studies in healthy participants, in whom central sensitization was induced with topical capsaicin and electrical LFS (Di Lionardo et al., 2021; Leone et al., 2023). Importantly, although electrical high frequency stimulation (HFS) also induces central sensitization in healthy participants, this protocol did not lead to a change in N13 amplitudes (Leone et al., 2023). The authors discussed this finding as LFS and HFS triggering central sensitization through different mechanisms: LFS through wind-up and heterotopic facilitation, while HFS may only induce heterotopic facilitation. These differences highlight that human experimental models, although valuable, capture only selected mechanisms of central sensitization and cannot replicate all aspects of a disease (Quesada et al., 2021). Furthermore, N13 SEPs were shown to be sensitive to top-down modulation, i.e., descending inhibition during a cold pressor test (Di Pietro et al., 2021). Although further studies regarding N13 SEPs are limited, evidence from other markers of spinal hyperexcitability, e.g., TSP (Rubal-Otero et al., 2024), noxious withdrawal reflex (Khatibi et al., 2014; Rhudy et al., 2018), and secondary mechanical hyperalgesia (Meyers et al., 2025), suggests that spinal excitability can also be modulated by expectations, emotions and attention.

Another possible explanation for the lack of increased N13 amplitudes in CRPS may be reduced afferent input at the peripheral level. While the cohort of CRPS type I is defined by the absence of a detectable nerve lesion, a recent study found decreased sensory nerve action potentials after stimulation of the median nerve of the affected, compared to the contralateral arm in CRPS type I (Yüksel et al., 2023). Notably, these decreased amplitudes were within a normal range. In our CRPS cohort the sensory nerve action potentials were also within range of normative data, and did not significantly differ between the two arms, nor compared to HC. Despite the absence of statistical differences between CRPS and HC in sensory nerve action potentials and the peripheral N9, these two components tended to be bilaterally smaller in the cohort of CRPS. This could potentially be explained by the increased weight of our CRPS cohort compared to HC, as obesity is likely linked to peripheral neuropathy (Elafros et al., 2024). Therefore, we cannot fully exclude a potential subclinical peripheral nerve impairment, which may have attenuated central neurophysiological processes. Alternatively, increased subcutaneous tissue associated with higher body weight may increase the distance between the neural source and the recording electrode, resulting in attenuated amplitudes of peripheral action potentials (Sand et al., 2025).

Taken together, the contribution of various underlying pathophysiological mechanisms to the phenotype of CRPS, including peripheral processes and psychological changes, may have limited the translation of findings from earlier experimental pain models to our clinical cohort.

### 4.2 The influence of medication on spinal excitability

The intake of diverse medication represents another potential contributor to the lack of differences in N13 amplitudes between CRPS and HC. Nine out of ten individuals with CRPS enrolled in our study were receiving one or more pain medications ranging from antiepileptics (i.e., pregabalin and gabapentin) to opioids (Table 1). The suppressing effect of pregabalin on N13 amplitudes has been previously described (Di Lionardo et al., 2021) and was linked to the pregabalin-related inhibition of postsynaptic neuron activation on dorsal horn level (Tuchman et al., 2010). However, the effect of other drugs on N13 SEPs is currently unknown and prompts us to focus on the effect of other drugs on secondary mechanical hyperalgesia, a hallmark sign of spinal hyperexcitability, and noxious withdrawal reflexes, another important readout for spinal excitability (Leone et al., 2025; Treede et al., 2022). For example, gabapentin was shown to decrease the area of secondary mechanical hyperalgesia after capsaicin sensitization in healthy participants (Dirks et al., 2002; Gottrup et al., 2004) as well as pinprick and brush allodynia in rat models of chronic pain (Field et al., 1997, Field et al., 1999), possibly through inhibitory effects on postsynaptic dorsal horn neurons (Rose and Kam, 2002). In case of opioids, a recent review found inconclusive effects of opioid receptor antagonist on the magnitude of mechanical hyperalgesia and the area of mechanical hyperalgesia after experimental pain models (Bedwell et al., 2025). However, opioids can also interact with other drugs on sensitization processes, as shown by supra-additive effect (i.e., a combined effect greater than the sum of each drug alone) on the reduction of area of hyperalgesia through the combination of tramadol and acetaminophen (Filitz et al., 2008). In addition, opioids, i.e., morphine, were shown to depress noxious withdrawal reflexes (Bossard et al., 2002; Willer, 1985; Willer and Bussel, 1980). Furthermore, antidepressants like serotonin noradrenalin reuptake inhibitors act on the level of the spinal dorsal horn by enhancing the activation of alpha-2-adrenergic receptors, leading to reduced neurotransmitter release from primary afferents and decreased excitability of second-order neurons (Obata, 2017). Taken together, it is plausible that the diverse pharmacological effects dampened dorsal horn excitability and thereby masked the hypothesized increase in N13 amplitude in CRPS. Despite these potential effects of medication on dorsal horn excitability, our data show increased QST values and TSP in the painful limb of CRPS. Whether the divergence between SEP and QST findings reflects a partial dissociation between neurophysiological and psychophysical measures of central sensitization (C. M. Leone et al., 2025), or arises from potential peripheral and/or supraspinal influences, remains to be elucidated.

### 4.3 Limitations

There are few limitations of this study worth mentioning. First, the small sample size of this study limits its statistical power and investigations of the N13 component in larger clinical cohorts are warranted. Second, due to the focus on spinal N13 SEP we only included individuals with CRPS affected at the upper limb. All included individuals with CRPS were female. Although women are affected by CRPS three to four times more than men, and the upper limb is more often affected (about 60%) than the leg (de Mos et al., 2007), our sample does not reflect the full CRPS population, which limits the generalizability of our findings. Third, peripheral nerve integrity was assessed only for the median nerve. Although all parameters were within normative ranges, assessment of additional nerves (e.g., radial nerve) could have further strengthened the exclusion of subtle or subclinical peripheral neuropathies. Fourth, while we are aware of the potential effects of medication on our readout, the small sample size and diverse medication intake did not allow for a subgroup analysis. Lastly, the relatively small amplitudes of N13 SEPs, together with limited normative data, and the absence of test-retest reliability studies, represent an inherent methodological challenge.

## 5 Conclusion

This is the first clinical study to investigate N13 SEPs as a marker of central sensitization in a chronic pain cohort. Despite clear signs of central sensitization in CRPS, including mechanical hyperalgesia and increased TSP, our findings showed no increased N13 amplitudes compared to age- and sex-matched HC. These findings challenge the utility of the N13 SEP component as a reliable marker of central sensitization in clinical pain cohorts. To implement N13 SEPs as a clinical readout, continued methodological refinement and validation in larger cohorts is needed.

## Declaration of competing interest

The authors declare that they have no known competing financial interests or personal relationships that could have appeared to influence the work reported in this paper.
