# Supplementary material for: The N13 somatosensory evoked potential in complex regional pain syndrome – A potential marker for central sensitization?
Source: Clin Neurophysiol Pract. 2026 Jul 1;11:551–9. doi: 10.1016/j.cnp.2026.06.013 (PMC13355545; doi:10.1016/j.cnp.2026.06.013)
Supplement: Supplementary file 1 — Supplementary material [file mmc1.pdf]

Table S1. Model statistics for outcomes of the NCS, QST, and multilevel SEPs.

	cohort		area		cohort * area	
variable	F-value	p-value	F-value	p-value	F-value	p-value
NCS amplitude	2.02	0.17	4.42	0.052	0.06	0.82
NCS NCV	0.70	0.41	1.57	0.23	8.30	0.01
HPT	10.71	0.004	14.65	0.001	16.36	<0.001
MPT	15.55	0.001	14.74	0.001	13.74	0.002
MPS	9.70	0.006	10.88	0.004	16.34	<0.001
PPT	7.92	0.01	16.86	<0.001	13.41	0.002
Stimulation intensity	0.02	0.90	2.66	0.12	0.02	0.90
N9 amplitude	2.86	0.11	2.03	0.17	0.72	0.41
N13 amplitude	0.57	0.46	0.01	0.91	0.42	0.53
N20 amplitude	0.2	0.66	0.63	0.44	0.03	0.86

Abbreviations: NCS: nerve conduction study; NCV: nerve conduction velocity;
SEP: somatosensory evoked potential; QST: quantitative sensory testing;
HPT: heat pain threshold; MPT: mechanical pain threshold;
MPS: mechanical pain sensitivity; PPT: pressure pain threshold

Table S2. Model statistics for outcomes of the TSP assessment.

	cohort		stimulation (1-3 vs. 10-12)		cohort * stimulation	
variable	F-value	p-value	F-value	p-value	F-value	p-value
TSP painful	2.93	0.1	76.11	<0.001	4.67	0.04
TSP contralateral	0.25	0.62	18.43	<0.001	0.006	0.94

Abbreviation: TSP: temporal summation of pain

Table S3. Performed post-hoc comparisons (R package 'emmeans').

variable	contrast	t-value	p-value	Hedges' g
NCV	CRPS pa vs. HC pa	-0.03	1.0	-0.01
	CRPS con vs. HC con	-1.57	0.25	-0.55
	CRPS pa vs. CRPS con	1.12	0.41	0.19
HPT	CRPS pa vs. HC pa	5.09	<0.001	1.89
	CRPS con vs. HC con	-0.03	1.0	-0.01
	CRPS pa vs. CRPS con	5.57	<0.001	1.82
MPT	CRPS pa vs. HC pa	5.38	<0.001	2.04
	CRPS con vs. HC con	0.36	0.49	0.47
	CRPS pa vs. CRPS con	5.34	<0.001	1.52
MPS	CRPS pa vs. HC pa	4.53	<0.001	1.90
	CRPS con vs. HC con	1.11	0.41	0.49
	CRPS pa vs. CRPS con	5.19	<0.001	0.95
PPT	CRPS pa vs. HC pa	4.56	<0.001	1.53
	CRPS con vs. HC con	-0.49	0.77	-0.35
	CRPS pa vs. CRPS con	5.49	<0.001	1.72
TSP pa	CRPS pre vs. CRPS post	7.70	<0.001	2.34
	HC pre vs. HC post	4.64	<0.001	1.29
	CRPS pre vs. HC pre	0.26	0.95	0.17
	CRPS post vs. HC post	2.61	0.03	0.90
TSP con	pre vs. post	8.72	<0.001	0.64

Abbreviations: NCV: nerve conduction velocity; HPT: heat pain threshold;
MPT: mechanical pain threshold; MPS: mechanical pain sensitivity;
PPT: pressure pain threshold; TSP: temporal summation of pain
CRPS: complex regional pain syndrome; HC: healthy controls;
pa: painful arm; con: contralateral arm

Table S4. Mean ± SD of amplitudes and NCV of the NCS.

area	cohort	NCS component		value
painful	CRPS	amplitude	N=10	15.2 ± 6.6 uV
		NCV	N=10	57.2 ± 3.4 m/s
	HC	amplitude	N=10	18.5 ± 5.9 uV
		NCV	N=10	57.2 ± 3.4 m/s
contralateral	CRPS	amplitude	N=8	13.7 ± 3.3 uV
		NCV	N=8	56.8 ± 3.7 m/s
	HC	amplitude	N=9	15.6 ± 3.9 uV
		NCV	N=9	59.0 ± 4.0 m/s

Abbreviations: SD: standard deviation; NCV: nerve conduction velocity; NCS: nerve conduction study; CRPS: complex regional pain syndrome; HC: healthy controls

Table S5. Mean ± SD of QST z-scores.

area	cohort	QST parameter		z-score
painful	CRPS	HPT	N=10	2.04 ± 1.12
		MPT	N=10	1.90 ± 0.88
		MPS	N=10	1.91 ± 1.25
		PPT	N=10	3.87 ± 2.74
	HC	HPT	N=10	0.22 ± 0.67
		MPT	N=10	0.42 ± 0.45
		MPS	N=10	0.05 ± 0.44
		PPT	N=10	0.61 ± 0.91
contralateral	CRPS	HPT	N=10	0.26 ± 0.62
		MPT	N=10	0.65 ± 0.57
		MPS	N=10	0.64 ± 1.19
		PPT	N=10	0.04 ± 1.10
	HC	HPT	N=10	0.27 ± 0.68
		MPT	N=10	0.40 ± 0.48
		MPS	N=10	0.18 ± 0.43
		PPT	N=10	0.39 ± 0.82

Abbreviations: SD: standard deviation; QST: quantitative sensory testing; HPT: heat pain threshold; MPT: mechanical pain threshold; MPS: mechanical pain sensitivity; PPT: pressure pain threshold; CRPS: complex regional pain syndrome; HC: healthy controls
